# Mapping biodiversity hotspots of fish communities in subtropical streams through environmental DNA

**DOI:** 10.1038/s41598-021-89942-6

**Published:** 2021-05-14

**Authors:** Rosetta C. Blackman, Maslin Osathanunkul, Jeanine Brantschen, Cristina Di Muri, Lynsey R. Harper, Elvira Mächler, Bernd Hänfling, Florian Altermatt

**Affiliations:** 1 Department of Aquatic Ecology, Eawag, Swiss Federal Institute of Aquatic Science and Technology, Überlandstrasse 133, 8600 Dübendorf, Switzerland; 2 Department of Evolutionary Biology and Environmental Studies, University of Zürich, Winterthurerstr. 190, 8057 Zürich, Switzerland; 3 Research Priority Programme Global Change and Biodiversity (URPP GCB), University of Zurich, Winterthurerstr. 190, 8057 Zurich, Switzerland; 4 Department of Biology, Faculty of Science, Chiang Mai University, Chiang Mai, 50200 Thailand; 5 Research Centre in Bioresources for Agriculture, Industry and Medicine, Chiang Mai University, Chiang Mai, Thailand; 6 Evolutionary and Environmental Genomics Group (EvoHull), School of Biological and Marine Sciences, University of Hull, Hull, HU6 7RX UK; 7 School of Biological and Environmental Sciences, Liverpool John Moores University, Liverpool, L3 3AF UK; 8 Department for Infectious Diseases and Pathobiology, Vetsuisse Faculty, Centre for Fish and Wildlife Health, University of Bern, Länggassstrasse 122, 3012 Bern, Switzerland

**Keywords:** Conservation biology, Freshwater ecology

## Abstract

Large tropical and subtropical rivers are among the most biodiverse ecosystems worldwide, but also suffer from high anthropogenic pressures. These rivers are hitherto subject to little or no routine biomonitoring, which would be essential for identification of conservation areas of high importance. Here, we use a single environmental DNA multi-site sampling campaign across the 200,000 km^2^ Chao Phraya river basin, Thailand, to provide key information on fish diversity. We found a total of 108 fish taxa and identified key biodiversity patterns within the river network. By using hierarchical clustering, we grouped the fish communities of all sites across the catchment into distinct clusters. The clusters not only accurately matched the topology of the river network, but also revealed distinct groups of sites enabling informed conservation measures. Our study reveals novel opportunities of large-scale monitoring via eDNA to identify relevant areas within whole river catchments for conservation and habitat protection.

## Introduction

Tropical and subtropical regions are among the most biodiverse systems globally^1,2^. Freshwater rivers in these regions are particular hotspots of biodiversity, with up to a third of global freshwater fish species found in the Amazon, Congo and Mekong river basins alone, most of which are endemic to these areas^3^. These extraordinary systems not only contribute strongly to global biodiversity, they also provide essential ecosystem services, such as drinking water and are a key protein source for local human populations^4^. The biodiversity within these systems is also essential to maintain their natural function and the services they provide^5^. However, tropical freshwater ecosystems and the biodiversity therein are subject to substantial anthropogenic pressures, such as change in land use, damming, direct exploitation of organisms, global climate change, pollution and the introduction of invasive species^5–8^. For example, dam construction and the loss of free-flowing rivers is causing excess siltation, barriers to fish migration and changes in hydrology, which is detrimental to the biodiversity and ecosystem services in these rivers^1,3,9,10^. These rivers are also notably more vulnerable to the effects of global climate change, with increased temperature and reduced flows likely to have significant effects on these systems in the immediate future^2,10^.

Conservation and management of tropical and subtropical freshwater systems depends on accurate, fast and reproducible knowledge on the state of biodiversity^11^. Often, however, even basic identification of biodiversity patterns within these systems are limited, due to their large scale or lack of monetary resources available^7^. Consequently, accurate baseline biodiversity information is generally non-existent or patchy in coverage, and little is known about the current distribution of species and the general status of biodiversity, even for charismatic groups such as fish^6^. Without this information, we risk being unable to identify community changes, species extinctions and, ultimately, biodiversity reduction at local, regional and global scales.

Identifying species distributions and patterns (specifically local and among-community diversity, i.e., α- and β-diversity, respectively) is the first step towards successful conservation^12^. It allows biogeographical clusters or regions to be established based on community data, and the designation of relevant conservation units. First described by Wallace in 1876^13^, this is a well-established and essential method in protecting key areas for conservation. Although division of land- and sea-scapes due to their assemblage of taxonomic groups (family, genus or species level data) is a common approach in ecological and evolutionary studies at global scales^14–16^, it also has direct application for regional and local conservation^16^. By identifying distinct clusters in continuous species distributions within a river catchment, it is possible to recommend priority areas for conservation at a finer spatial scale.

With the advent of environmental DNA (eDNA) metabarcoding, studies have quickly demonstrated its huge potential to monitor target taxa and communities on a landscape scale^17–19^. The approach is based on the isolation of DNA from water samples and subsequent High-Throughput-Sequencing of PCR amplified DNA barcodes. The selection of suitable PCR primers allows the identification of species from specific taxonomic groups present in an ecosystem^20^. This approach has been used to assess species richness of fish in different temperate aquatic systems^21–25^, providing crucial information for fisheries and conservation management^26^. So far, however, this has not yet been scaled to spatially very large, species-rich systems, and eDNA studies of fish within tropical and subtropical riverine ecosystems have focused mainly on the detection of individual species, using a species-specific approach (e.g.,^12,27–29^). Although eDNA metabarcoding has the potential for community-wide assessment, attempts to gather the much-needed baseline biodiversity data from tropical and subtropical hotspots have so far been limited^30–32^. By being highly scalable, non-invasive to the taxa being detected, and allowing prediction of diversity patterns across whole riverine systems^33,34^, it is offering an unprecedented opportunity to monitor freshwater habitats on a landscape scale.

**Figure 1:**
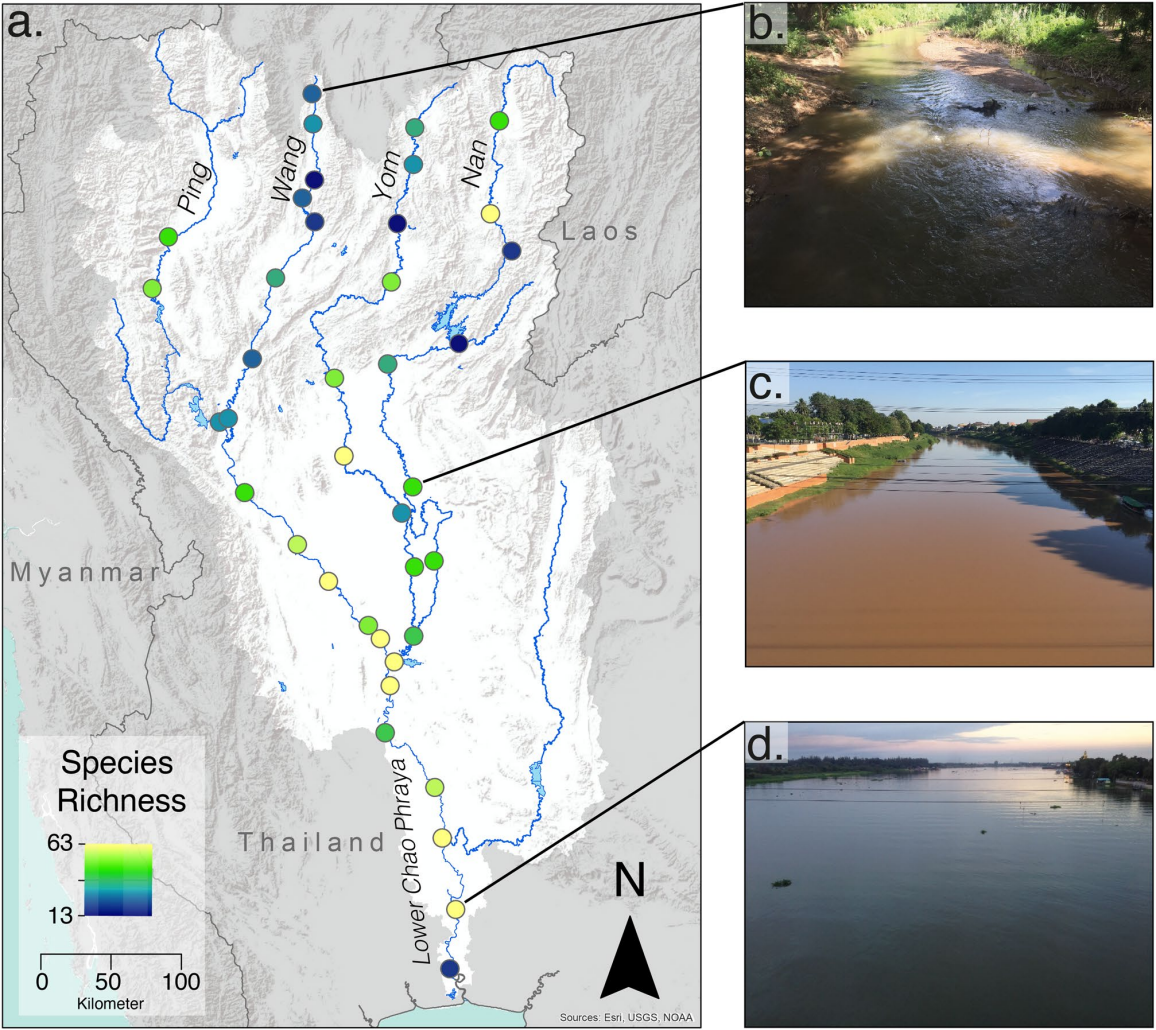
Local species richness of fish collected from eDNA samples in the Chao Phraya catchment and representative site illustrations. (**a**) Local species richness (α-diversity) of freshwater fish in the Chao Phraya catchment (outlined by the white background) located in Northern Thailand, (**b**) Channel photograph at a headwater stream (site 9: River Wang), (**c**) Channel photograph at a mid-order stream (site 31: River Nan), (**d**) Channel photograph at a lowland stream near Bangkok (at site 38: lower Chao Phraya river). Approximate cross-section width at these sites is 9 m, 75 m, and 250 m, respectively. The base map data was sourced from HydroSHEDS. 2015: WWF in partnership with USGS, CIAT, TNC, CESR: Esri, 2013 and mapped using ArcGIS. Photographs provided by M. Osathanunkul.

Here, we use eDNA metabarcoding to assess fish diversity patterns in a global biodiversity hotspot the Chao Phraya catchment, Thailand. This catchment comprises four tributaries: Nan, Ping, Wang and Yom, which combine to form the main Chao Phraya river (Fig. 1a and Supplementary Information Fig. S1). The catchment covers over 200,000 km^2^. Only limited biodiversity surveys have focused on the middle and lower reaches of the catchment, where a high number of threatened and endemic freshwater fish species have been recorded, therefore it represents a major centre of species richness^6^. As with many rivers in Asia, the Chao Phraya is under increasing anthropogenic pressures and remains poorly surveyed^6,35^. We extracted DNA from 234 water samples collected from 39 sites across the basin. We analysed the eDNA for fish communities using two primer pairs which amplify different sections of the mitochondrial 12S region and are vertebrate-specific (Kelly primer pair)^36,37^, and fish specific (MiFish primer pair)^38^ respectively. Amplicons were sequenced on the Illumina MiSeq platform, and sequencing data were bioinformatically cleaned and assigned to 249 reference sequences of fish species (see “Methods” section). Although the distribution of species without reference sequences cannot be evaluated with this approach, our reference database comprises 54% of all fish species known or suspected to be present in the Chao Phraya river basin and includes species from most families and ecological guilds. We thereby captured an unprecedented and unbiased fingerprint of the freshwater fish diversity within the catchment and identified possible areas of conservation importance, proving that acquisition of biodiversity data, even at large scales, shall no longer be a limiting factor for the assessment and implementation of biodiversity-conservation measures in tropical and subtropical lotic systems.

## Results

After bioinformatic clean-up and removal of low frequency reads, across all samples a total of 5,825,212 and 4,927,576 reads were assigned to Fish Taxa (hereafter FT, see “Methods” section) found in the custom-built reference database for Kelly and MiFish primers, respectively. On average 149,364 and 126,348 reads were assigned to each of the 39 sites sampled for the Kelly and MiFish libraries, respectively. From the two libraries, we successfully detected 108 distinct FT: 82 with the Kelly primer and 93 with the MiFish primer. Of the 108 FT found in the combined dataset, 67 (62%) FT were found with both primer pairs, 15 (14%) were unique to the Kelly dataset and 26 (24%) were unique to the MiFish dataset. The detection of FT unique to one primer dataset was in some cases due to the lack of reference sequences for one of the two marker regions. The Kelly primer detected only one FT not present in the MiFish reference database, and the MiFish primer detected six FT not in the Kelly reference database (see Supplementary Information Table S2 and S3). Overall, there was a highly significant correlation between read abundance generated by the two primer sets for each FT in the datasets (R = 0.37, *p* = 0.002, see Supplementary Information Fig. S2). To examine α- (local) and β- (among-community) diversity, we combined all six replicates taken at each site (two replicates from each site location at right bank, centre and left bank, respectively (see Supplementary Information Fig. S3 for proportion of taxa found in each replicate), and converted the data to presence/absence.

**Figure 2:**
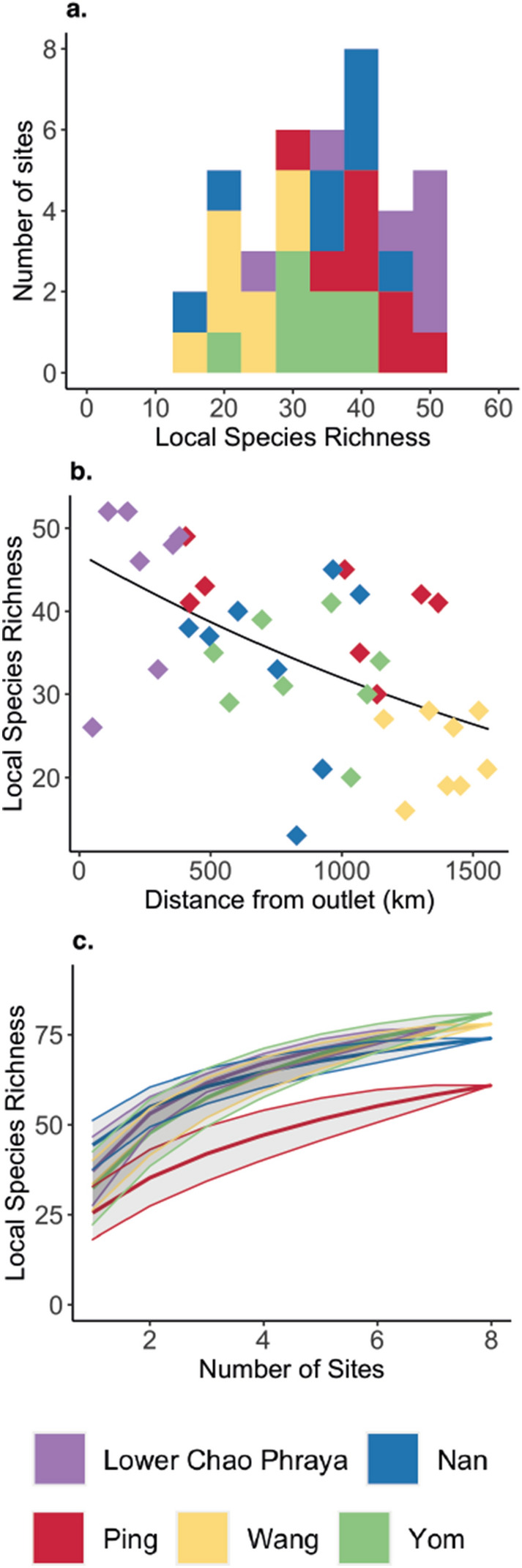
Patterns of local species richness: (**a**) frequency distribution of local species richness over the 39 sampling sites in the Chao Phraya catchment, (**b**) Local Species Richness as a function of the river distance from the outlet (the black line is the GLM prediction), and (**c**) Local Species richness accumulation curves from each river (mean and standard deviation). Colours represent each rivers: Lower Chao Phraya (lilac), Nan (blue), Ping (red), Wang (yellow), and Yom (green). Figure produced using R studio, version 3.6.3 with the ggplot2 package.

Local richness (α-diversity) ranged between 13–52 FT (Figs. 1a, 2a). Sites sampled (Fig. 1c–d for illustrations) are representative of sites along the backbone of the total catchment covering 200,000 km^2^ in Northern Thailand, and included streams varying in width from 8 to 250 m. We identified distinct α-diversity patterns, with local richness in headwaters (Ping, Wang, Yom and Nan) being overall lower than in the downstream section of Chao Phraya. This is corroborated by a highly significant decrease in local richness with increasing topological distance from the river outlet at the Gulf of Thailand (Fig. 2b, GLM, z = 68.925, *p* < 0.001, AIC 303.48). Species richness accumulation curves for all five rivers have a more steeply increasing slope initially and begin to plateau after about five to six sites sampled (Fig. 2c), with the local richness starting to plateau for the River Ping before the other rivers (see Supplementary Information Fig. S4 for total species richness accumulation for the catchment).

**Figure 3:**
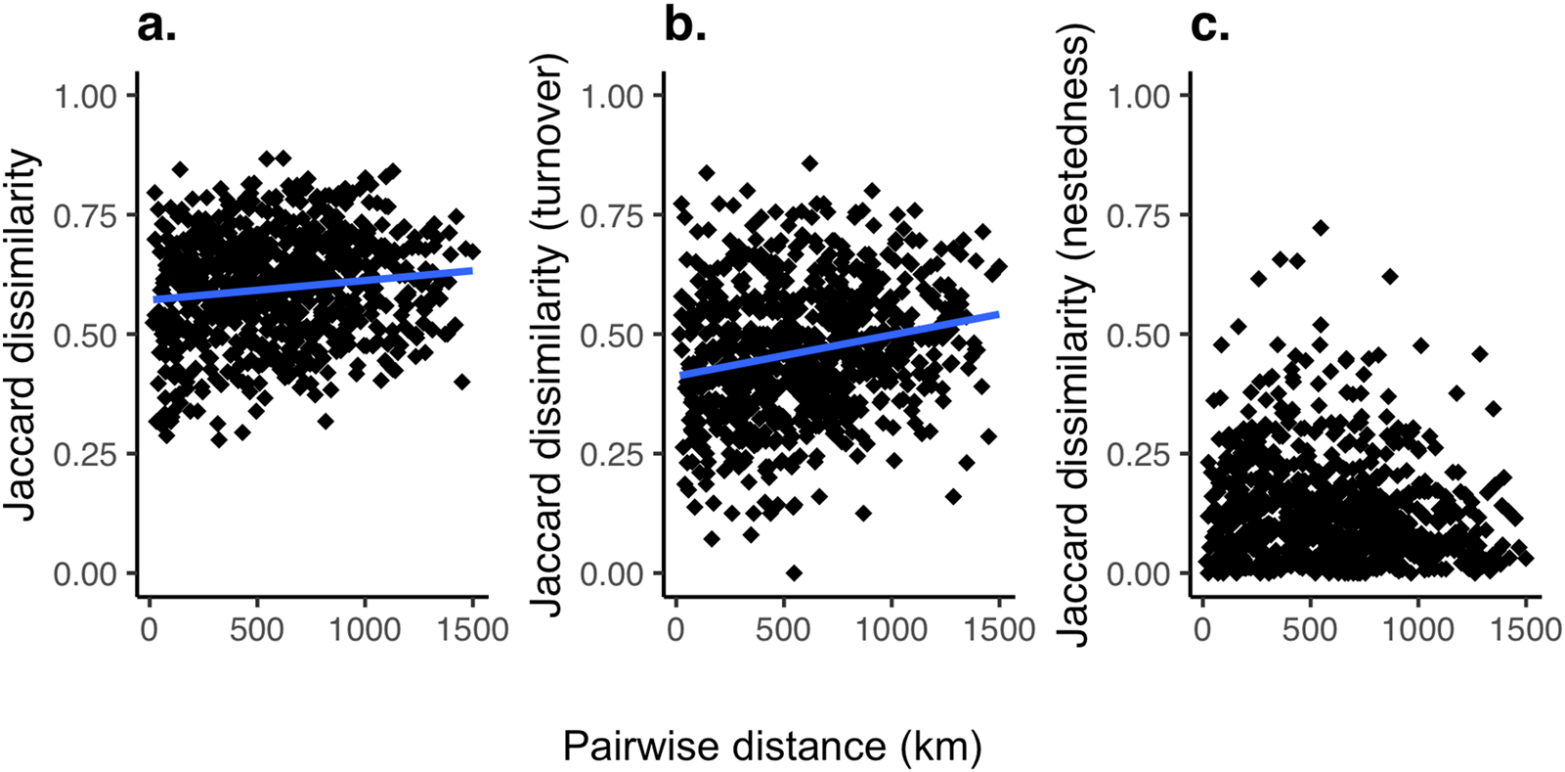
Pairwise β-diversity against river distance for all sites. β-diversity is calculated based on Jaccard dissimilarity where 0 would indicate identical communities. The blue line indicates the significant correlation using Mantel statistic: (**a**) Jaccard dissimilarity, Mantel statistic: 0.15, *p* < 0.05, (**b**) turnover, Mantel statistic 0.23, *p* < 0.005 and (**c**) nestedness, not significant. Figure produced using R studio, version 3.6.3 with the ggplot2 package.

To assess community change throughout the river network, we used Jaccard dissimilarity as a measure of β-diversity (Fig. 3a). Jaccard dissimilarity can be further partitioned into taxon replacement (turnover, Fig. 3b) and taxon loss (nestedness, Fig. 3c). These measurements allowed us to assess the variation in community assemblage across the catchment^39^. A Mantel test of Jaccard community dissimilarity versus geographic distance along the river network concluded both Jaccard’s dissimilarity and turnover significantly increased as a function of increased river distance (Mantel statistic: 0.15, *p* < 0.05 and 0.23, *p* < 0.005, respectively), while Jaccard’s nestedness or taxon loss did not statistically increase as a function of river distance (Mantel statistic: − 0.1479, *p* = 0.977).

**Figure 4:**
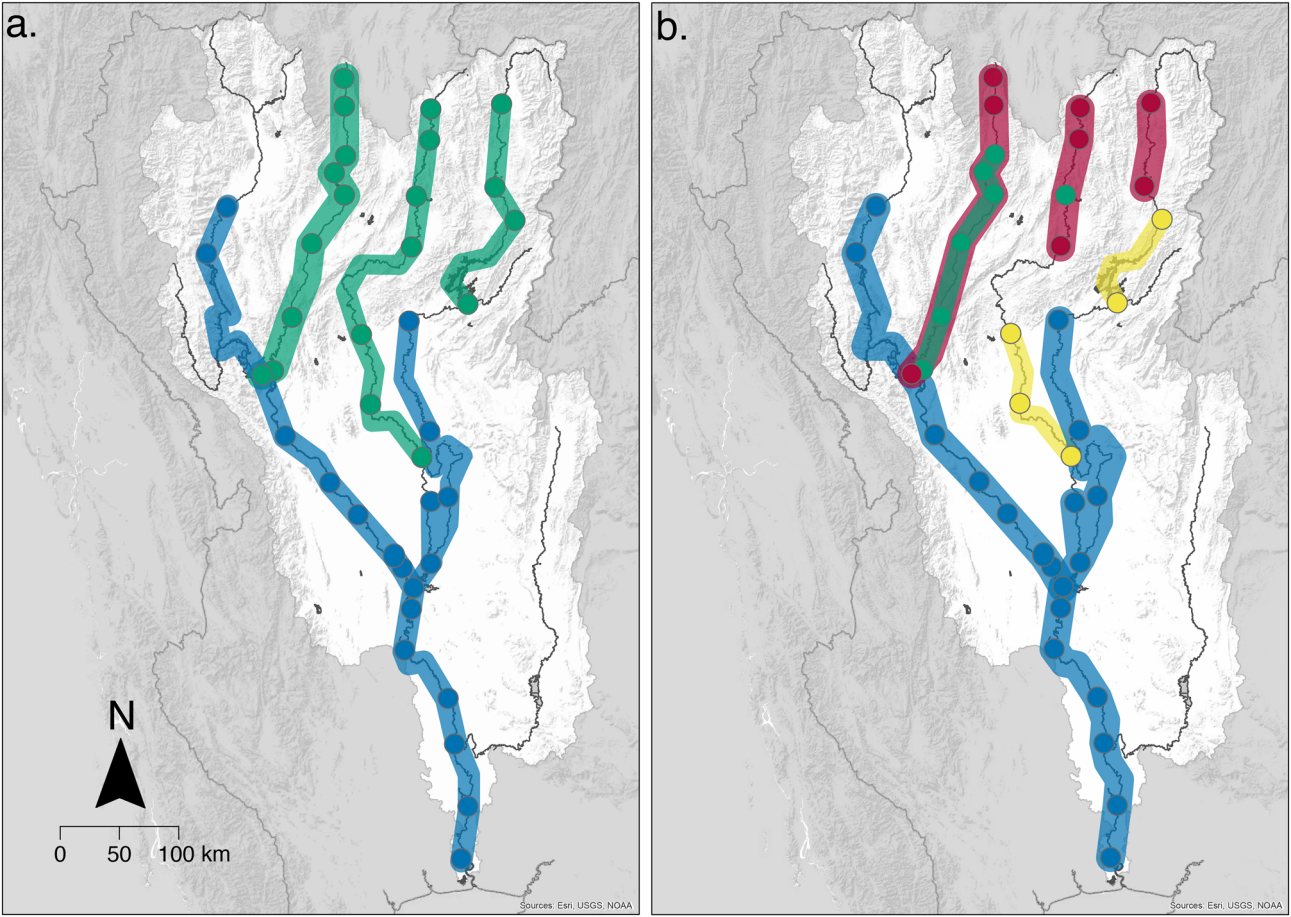
Biogeographical conservation units. Maps illustrating the projections of the optimal clusters, plotted across the Chao Phraya catchment to visualise the fish community in space. Clusters are joined with associated cluster colour where sites are connected by downstream flow. Optimal cluster validation measures supported both two (**a**) and four (**b**) clusters. The base map data was sourced from HydroSHEDS. 2015: WWF in partnership with USGS, CIAT, TNC, CESR: Esri, 2013 and mapped using ArcGIS, coloured clusters were added using Adobe Illustrator version 25.2.1.

Together with α-and β-diversity measures, the similarity of community assemblages within a catchment is an important aspect in identifying key biodiversity patterns within an ecosystem. To consider the importance of these biogeographical conservation units, we examined the read data generated by taking the maximum read count for each FT detected at each site. Of the clustering methods we compared, hierarchical clustering had the most support and generated two optimal number of clusters dependent on the cluster validation measure (see Table 1 and Methods section for further details). Two clusters were supported by three validation methods: Connectivity, Dunn Index and Average Distance between means. Four clusters were supported by two validation methods: Silhouette coefficient and the Average proportion of non-overlap. To investigate the clusters further, dendrograms for each optimal cluster number (two and four) were plotted (see Fig. S5) and clusters were projected on the Chao Phraya catchment (see Fig. 4a,b).

**Table 1:** Optimal cluster validation measures: internal and stability measures show the most support for hierarchical clustering methods (5 out of the 7 measures tested), with support for 2 and 4 clusters (see Fig. S6).

Type of measure	Measure	Optimal cluster method	Optimal number of clusters
Internal	Connectivity	Hierarchical	2
Internal	Dunn index	Hierarchical	2
Internal	Silhouette width	Hierarchical	4
Stability	APN	Hierarchical	4
Stability	AD	PAM	10
Stability	ADM	Hierarchical	2
Stability	FOM	PAM	7

When projecting the clusters across the river network based on two optimal clusters (the optimal cluster number with the greatest support by validation measures), there is a clear spatial separation of two major clusters across the catchment (Fig. 4a). The Wang, Yom and headwaters of the rivers Nan closely cluster, which can also be considered upland habitat (hereafter, referred to as the North East cluster). The River Ping, main Chao Phraya channel and lower part of the Nan are a second distinct cluster (hereafter, referred to as the South West cluster).

When projecting four optimal clusters (also supported by the validation measures), we see the North East cluster is divided further into three clusters. Subsequently, greater details of the underlying communities present emerge, which is indicative of the distinct communities found at the highest points in the catchments within the original North East cluster (Fig. 4b). The red cluster is formed predominantly of the high-altitude sites of the Rivers Wang, Yom and Nan, while the yellow cluster is made of sites found in the low and middle reaches of the Rivers Yom and Nan.

Overall, clear and distinct clusters emerge, but some spatial overlap of different clusters can be seen in the projection of four clusters (Fig. 4b, red and green clusters), and is restricted to sites within Rivers Wang and Yom. This spatial coherence strongly supports the adequacy of the data, as some overlap would naturally be expected both due to eDNA transportation and the community turnover occurring along distinct zones of transitions, which are expected to stretch over some distance. In contrast, the entire River Ping and Chao Phraya are consistently considered as a single cluster in both projections (Fig. 4a,b) and this grouping can also be seen in a principal component analysis of the environmental variables for these two rivers (Fig. S7a).

## Discussion

Tropical and subtropical freshwater systems face unprecedented anthropogenic threats from increased habitat modification, exploitation of fisheries, global climate change, pollution and invasive species^5–7^. Our study focused on such an ecosystem, the Chao Phraya catchment in Thailand, which is a global hotspot for fish diversity, including many endemic and threatened species^6^. Like most other rivers in tropical and subtropical South-East Asia, current, accurate and spatially comprehensive biodiversity data to underpin evidence-based environmental policies is lacking^11^. Here, we used a novel approach which could easily be applied to other comparable ecosystems across the globe to describe spatial biodiversity patterns and define conservation priorities. By conducting a single eDNA survey of water samples collected from 39 sites, we identified 108 freshwater FT and distinct clusters of species assemblages within the river network. Our data shows clear α-diversity accumulation towards the outlet of the catchment, increased β-diversity as a function of river network distance and separation of sites into distinct biogeographically distinct clusters. These three key elements of biodiversity patterns in the Chao Phraya catchment have strong implications for conservation. Specifically, they align with previous studies and provide the necessary data showing that headwaters harbour a distinct community of fish species^40,41^ and should be preserved for their unique composition, while sites further downstream and in the lowlands support a higher richness and should be considered for conservation of maximal number of species^42,43^. While such data are generally available for temperate regions (e.g.,^42^) and predicted by theoretical models (e.g.,^44,45^), adequate data supporting necessary environmental decision making has hitherto been largely lacking for tropical and subtropical regions but can now be achieved as shown here.

By assessing the α-and β-diversity at the level of functional taxonomic units, we are able to determine fundamental properties of the fish diversity at a catchment scale. Species richness is often seen as the most basic and vital component of an ecosystem’s productivity and function^46^. In the Chao Phraya region, α-diversity ranged from 13 to 52 FT across the catchment, with a significant increase in diversity towards the outlet. Likely, the total numbers are an underestimation due to the lack of complete genetic reference databases, but by using eDNA we have captured a sufficient subset of the diversity to obtain meaningful results, which are consistent with ecological theory. Furthermore, we identify particular species-rich areas in the middle and lower parts of the catchment, directly offering conservation targets^6^ (Figs. 1a, 2a). Compared to other studies, our data demonstrate these patterns on unprecedented and biogeographically relevant landscape scale, rather than at a local perspective^23,47^. In terms of key β-diversity patterns, Jaccard’s dissimilarity and turnover increase significantly with river distance, as found in other large-scale studies on fish communities^23,42,43^, while nestedness or species loss was not significant. This is indicative of the high fish diversity within the catchment and likely matches the varied habitats types in the catchment^39^. The lack of nestedness also suggests that our data are not likely to be significantly affected by eDNA transport between adjacent sites, which theoretically could result in inflated diversity estimates at downstream sites. In lotic ecosystems, local eDNA signals provide a view of the community upstream of the sampling site^18,34^. The exact spatial scale over which the signal is integrated depends on the environmental conditions, but most previous studies in temperate regions have shown that the signal degenerates rapidly and detection can be up to 10 km away from the source (summarised in^24^). However, one study, based on filtration of 30 L of water found transportation in large temperate rivers could occasionally be further than 100 km, but even in this case the effect on the estimation local biodiversity patterns was low^24^. In our study, sample sites were on average 72 km apart and we filtered a relatively small volume of water per site, with environmental conditions unfavourable for DNA persistence (i.e., during the dry season). Hence eDNA transportation is unlikely to affect our conclusions. Future studies should combine this information with models of eDNA transport (e.g.,^34,48^), in order to understand the persistence and degradation of eDNA across temporal sampling and improve our knowledge of where and when to sample, and further support this method for fish diversity assessment over large scale catchments.

By using biogeographical clustering, we identified transitions in fish community structure across the Chao Phraya catchment, allowing us to identify areas for protection of relevant and important community assemblages. The two-cluster solution separated the catchment into two geographically distinct regions in the North East and South West, respectively (Fig. 4a). These clusters also differ in key ecosystem characteristics and is demonstrated further by the PCA and grouping of river sites in Fig. S7a. The North East cluster comprises high to medium altitude smaller river sites compared to the larger lowland rivers in the South West cluster. We would therefore recommend that conservation practices within the Chao Phraya catchment ensure representative sites from each of these two strongly supported optimal clusters are included in order to protect the two distinct communities. Being able to separate the sites into clusters is fundamental to identify areas of conservation priority^49,50^. The four-cluster solution indicates a fine-scale sub-structuring of the North East cluster, whereas the South-West cluster remains a single cluster (Fig. 4b). This supports the idea that the fish communities in the lowland regions (River Ping and main Chao Phraya) are more homogenous compared to the upland tributaries, which might require a more nuanced conservation strategy. This projection separates the North East cluster into three further clusters, of which two are geographically distinct from each other align with different eco-regions within the catchment, the upper reaches (Fig. 4b—red cluster) and middle reaches (Fig. 4b—yellow cluster). Although such a zonation is theoretically predicted by the river continuum concept and through the gradual change of co-varying environmental characteristics^46,51,52^ (as seen in Fig. S7, which shows river site groups from environmental variables recorded at each site), our data crucially provides the information for the further demarcation of these zones, a prerequisite for identifying areas to focus conservation effort on within a large area. This is also important due to marked vulnerability of upstream regions to global climate change, increase in temperature and the associated reduced flow patterns and connectivity, compared to the lower reaches of the catchment^2,46^. The four-cluster projection also identified a cluster (Fig. 4—green cluster) with no clear geographical distinction, largely overlapping with two other clusters in the middle reaches of the rivers Wang and Yom (Fig. 4b). This indicates that additional ecological variation exists within these regions beyond the gradual zonation along the river continuum. This might not be captured adequately within the current sampling design and additional eDNA studies on a smaller geographical scale are warranted to investigate this further. Transport of the eDNA might theoretically also contribute to such an admixed community signal. However, given the spatial scale of our study and the absence of significant nestedness in the β-diversity pattern, it seems unlikely to be the predominant factor. Therefore, we can assume that the overlapping clusters may indicate transitional zones of species distribution within the two rivers, which also strengthens the need to protect sites in both clusters (red and green) due to their differing fish assemblages. Overall, the projected clusters (two and four) reveal distinct geographic areas of species assemblages allowing protection of relevant and important community assemblages across an ecosystem.

By matching sequences from our data with known fish species in Thailand, we were able to establish distinct communities of FT. These patterns could be detected through a surrogate approach, using an unbiased well-defined subset of the total fish diversity. Incomplete species detection is common for fish sampling approaches such as electro-fishing and gill-netting, but which species have been missed through such methods is difficult to predict. In contrast, we have clear predictions from our in-silico database analysis as to which species we can detect with the eDNA metabarcoding approach. Additionally, numerous studies have shown that false negatives rates are otherwise considerably lower for fish eDNA metabarcoding compared to all conventional methods^21,22,24,26^. We are therefore confident that our results are meaningful in the context of determining spatial biodiversity patterns. Nevertheless, increasing the comprehension of the database would provide new opportunities for more detailed ecological studies. For example, none of the databases provided reliable target sequences of the widespread fish genus *Schistura*^6^, which a priori excluded taxa within this genus from being detected. Complete databases would also allow detection of those species which were rare and endemic to the Chao Phraya catchment and aid our identification of specific species driving clusters. As with other eDNA studies (e.g.,^30,32^), specialist curated reference databases are key in highly diverse systems, but paradoxically are lacking due to the limited understanding of these ecosystems. Our approach of identifying biodiversity hotspots using eDNA can guide the targeted search for species, eventually benefiting the complementation of reference databases.

Of the taxa detected with the two markers, a number were only identified with one of the two 12S primer pairs. Some of these differences were caused by an unequal coverage of the reference databases for both primers, that is 20 of the 26 and 14 of the 15 FT were not detected with Kelly and MiFish, respectively, despite a reference sequence being present in the respective reference database and the FT being detected with the other primer pair. Possible, but not mutually exclusive, explanations for such imperfect detections include database errors, primer bias and differential resolution at species level^21,30,32^. In our case, all sequences used to curate a custom database were mined from public databases without the possibility of independent verification of the record. To exclude potentially misidentified records, phylogenetic placement methods were included in the curation, but these methods are not able to identify all errors, especially when the underlying phylogeny is incomplete. A single incorrect record for one of the two databases could potentially result in a discordance of two species between the two data sets. The highly diverse fish fauna investigated here contains many closely related species and given the short marker fragments used in this study, it is likely that a number of species pairs cannot be resolved, resulting in sequence assignments to a higher taxonomic level, which in most cases would have resulted in a loss of the associated taxa. Although the limited coverage of our database precludes a meaningful *in-silico* analysis, our results show that a significant proportion of sequences (24%) were assigned to the genus or family level lending support to this hypothesis. Primer bias, that is the differential amplification of individual species largely due to mismatches in the primer binding region, is often used as the default explanation for false negatives in metabarcoding studies, especially when primers target a broad taxonomic scale, for example, eukaryotes^18,53^. However, this is less likely to be a major factor in markers with a more limited taxonomic scope, such as the vertebrate and fish specific assays used here. *In-silico* and *in-vitro* analysis for these markers in other regional faunas have also shown little evidence of significant amplification bias^21,54^, although the possibility for such biases might be increased in highly diverse fauna investigated here. All of these issues can be addressed in future studies by improving the coverage of the reference database, using a larger number of markers and increased replication in various stages of the experimental design.

## Conclusion

In this study, we demonstrated that eDNA metabarcoding is a highly efficient way to generate data at the necessary scale required to map and assess biodiversity across large catchments of highly diverse but understudied tropical and subtropical freshwater systems. The data from this study were generated from water samples taken in a single sampling campaign across seven weeks, an effort which can easily be upscaled to provide finer geographical resolution and to cover seasonal fish migration. With such a moderate effort, we generated data that allowed us to identify key biodiversity patterns on a catchment scale and outline conservation priorities. This has hitherto been mostly prohibited due to the lack/difficulty of access to appropriate data. Collecting such data based on conventional methods and in a consistent way is simply too costly and time-consuming, hampering conservation effort for many biodiversity hotspots of global importance. Environmental DNA based approaches are a solution to this problem, and we believe that concerted efforts must now be made to harness this potential and ultimately prevent further loss of biodiversity.

## Methods

### Sampling and extraction

This study was conducted in Thailand in November 2016 (during the dry season, i.e., base-line low-flow conditions). Water samples were collected at 39 sites (Fig. 1a and Supplementary Information Fig. S1) along the main river network of the Ping, Wang, Yom, Nan and Chao Phraya rivers in Thailand over the course of 7 weeks (mean river distance between sites 72.5 km ± 6 (SE), range 16–184 km). At each site, water was sampled at three locations across the river width: left bank, centre and right bank (mean river width 112 m ± 15 (SE), range 9–361 m). This was done by either accessing the river channel from a bridge or with a small boat. At each of these three locations, two replicates were taken (i.e., in total six replicates were taken per site, resulting in n = 234 water samples taken). Water was collected by submerging a sterilized plastic bucket (rinsed with 10% chlorine bleach and deionised water after each sampling to avoid cross-contaminations) in the river and then subsampling the water with a 60 mL syringe. Per replicate, 100 mL of water was filtered through an enclosed 25 mm diameter, glass fibre filter membrane with 0.7 µm pore size (Whatman, GE Healthcare, UK) to collect all DNA from the water, resulting in a total of 600 mL of filtered river water per sampling site. At the start and end of each day, 300 mL ddH_2_O was filtered in the same manner to act as negative controls. At each site, river width (m), average pH, average conductivity, total dissolved solids and altitude (m) were recorded, and these environmental variables were analysed using a Principal Component Analysis (PCA) (See Supplementary Information Fig. S7a). Filters were placed in individual 1.5 mL Eppendorf tubes and kept in a polystyrene box containing dry ice before transferring to a − 20 °C freezer until extraction. All samples were extracted within 2 days of sampling in a dedicated clean laboratory, following the DNeasy Blood and Tissue Kit (Qiagen, Hilden, Germany) with a minor modification (see^46^). Samples were eluted in 100 µL of TE buffer and subsequently preserved at − 20 °C. In addition, each sample (n = 234) was treated with the OneStep PCR Inhibitor Removal Kit (Zymo Research) to remove potential PCR inhibitors bound to eDNA molecules that may be present in water samples.

### PCR, library preparation and sequencing

Samples were amplified using two 12S markers: a 106 bp fragment (forward primer sequence: 5′-TACTGGGATTAGATACCCC-3′ and reverse primer sequence: 5′-CTAGAACAGGCTCCTCTAG-3^36,37^) hereafter known as the Kelly primer and the MiFish-U-F/R primer pair (forward primer sequence: 5′-GTCGGTAAAACTCGTGCCAGC-3′ and reverse primer sequence: 5′-CATAGTGGGGTATCTAATCCCAGTTTG-3^38^) hereafter known as the MiFish primer pair, which targets a hypervariable region of the 12S rRNA gene which amplicon ranges from 163 to 185 bp. In brief, both libraries were prepared using the Illumina MiSeq dual-barcoded two-step PCR amplicon sequencing protocol, where the DNA was amplified using primers with an overhang in the first PCR, and in second reduced cycle PCR Nextera XT Indexes were added in order for samples to be identified during bioinformatic processing. The first PCR reaction contained 0.5 μM each primer, 0.4 mg/mL BSA, 12.5 μL Q5 High Fidelity 2X Master Mix (New England Biolabs), and 2 μL template DNA for both libraries. PCR profiles were as follows: for the Kelly library—initial denaturation 98 °C for 5 min followed by 35 cycles of 98 °C for 10 s, 58 °C for 20 s, and 72 °C for 30 s, and a final extension step of 72 °C for 7 min. In case there were double bands for the amplicon observed, the elongation time was decreased to 10 s. MiFish library—initial denaturation of 98 °C for 5 min, followed by 35 cycles of 98 °C for 10 s, 65 °C for 20 s, and 72 °C for 30 s, and a final extension step of 72 °C for 7 min. The first PCR was carried out in triplicate and samples were pooled and cleaned using SPRI beads (Applied Biological Materials Inc.) prior to the second PCR. Second PCRs were carried out using 15 μL of cleaned PCR product and the Nextera XT Index Kit v2 (Illumina), following the subsequent PCR profile: initial denaturation 95 °C for 3 min followed by 10 cycles of 95 °C for 30 s, 58 °C for 30 s, and 72 °C for 30 s, and a final extension step of 72 °C for 5 min. Negative controls and positive controls (a synthetic DNA sequence, see Supplementary Information Table S1) were processed in parallel with all samples. All PCR products were visualised using QiAxcel Advance System by using a High Resolution Cartridge (Qiagen, Hilden, Germany) and cleaned once more using SPRI beads. Samples were then quantified using the Spark 10 M Multimode Microplate Reader (Tecan Group Ltd.) using the Qubit dsDNA BR assay (Thermo Fisher) and pooled equimolar. The libraries were loaded at 15 pM concentration, with 10% PhiX control. A paired-end 300 cycle (2 × 150 nt) sequencing was performed on an Illumina MiSeq (MiSeq Reagent Kit v3, 200 cycles) following the manufacturer's run protocols (Illumina).

### Reference database and bioinformatics

In order to ensure sequences were assigned correctly, we created a reference database of fish known to occur in Northern Thailand. Firstly, we listed all freshwater fish recorded in Thailand according to (1) OEPP Biodiversity Series Vol. 4 Fishes in Thailand^55^ and (2) the Checklist of Freshwater Fishes of Thailand (www.siamensis.org). In total 408 species, including known invasive non-native species were checked and searched against GenBank in order to create a reference library of 12S sequences that could be used to assign our eDNA sequences too (see Supplementary Information Table S3). We found sequences for a total of 220 species (54%), which then were used as our reference library (see Supplementary Information Table S3). Importantly, and as commonly the case for such studies^56^, the incomplete database restricts the number of species that can be detected by our approach. We note that the absence of species in the genetic reference database is not random across the fish community. Large and charismatic species are more commonly represented, while smaller, or highly diverse species groups (e.g., some genera, such as *Schistura,* are underrepresented in the database or even lack completely). Processing of Illumina reads data and the taxonomic assignment were performed using a custom bioinformatics pipeline incorporating open source software (metaBEAT, v.0.97.7-global) as described in Hänfling et al.^21^ with the addition of the reference database as described above, using a threshold of 97% similarity for taxonomic assignment.

### Data analysis

Taxonomy of fish within Thailand is still very heterogeneous, with many databases not being very reliable, and no common agreement on taxonomic or faunistic databases existing (pers. comm. Maurice Kottelat). We thus decided to restrict our data analysis to fish taxa (FT) derived from those reads assigned without using taxonomic nomenclature. This approach may be ideal for tropical and subtropical catchments, which have a high diversity of organisms but often low or infrequent monitoring activities and therefore inadequate access to curated reference databases or the ability to make them.

The following thresholds were applied in order to minimise any potential contamination caused during laboratory work: 0.1% of total reads were removed from each sample, in line with similar studies^21,57^. For greater stringency, the greatest number of reads for each taxon found in the negative controls was also removed from each sample. To examine α-and β-diversity patterns, we combined the two primer data sets (Kelly and MiFish) and the biological replicates from the three sampling locations at each site (right bank, centre and left bank). Data was then converted to presence/absence. We calculated local species richness (α-diversity) at each site and used the glm() function with a Poisson distribution to analyse the effect of topological distance on species richness. Topological distance was measured using the measurement tool available on GoogleMaps. To compare β-diversity between sites, we used Jaccard dissimilarity partitioned into nestedness and turnover with the R package “betapart”^39^. We constructed a matrix of pairwise distances between sites along the fluvial network with the R package “igraph”^58^. To compare the similarity between β-diversity and river distance, we carried out a Mantel test with the R package “vegan”^59^. To examine FT richness accumulation within each river, we plotted species accumulation curves using the accumcomp() function in the R package “BiodiversityR”^60^.

To distinguish biogeographical units (unique and important sites or clusters of sites) within the catchment, we used the read count as a proxy for taxa signal within the catchment. We combined the two datasets (Kelly and MiFish) by taking the maximum count from each taxon of either dataset at each site. This enables us to reflect the full community assemblage taking into consideration the primer bias of each primer set (i.e., species not detected equally well by both primers). Firstly, we used Hopkin’s statistics, where 0 indicates uniform data and 1 highly clustered data to test if our data was suitable for clustering, we found our data was suitable for clustering (0.74). We compared clustering methods (hierarchical, k-means, Divisive ANAlysis (DIANA), Partitioning Around Medoids (PAM) and Self-Organising Tree Algorithm (SOTA)) and optimal cluster number using R package “clValid”^61^. For optimal cluster validation, we calculated Internal and Stability measures: Connectivity, Dunn Index, Silhouette coefficient, Average proportion of non-overlap (APN), Average Distance (AD), Average Distance between means (ADM) and Figure of Merit (FOM). Hierarchical clusters were generated using “ward.D2” linkage function and Canberra distance. Dendrograms were produced and explored using the R packages “stats”^62^ and “factoextra”^63^, and clusters were mapped on to the catchment using Adobe Illustrator. Base map data was sourced from HydroSHEDS. 2015: WWF in partnership with USGS, CIAT, TNC, CESR: Esri, 2013 and mapped using ArcGIS. All statistical analyses were performed using R studio, version 3.6.3^62^.

## Supplementary information

Supplementary information.

## Data Availability

Sequencing data is deposited in the European Nucleotide Archive under the primary accession number PRJEB34331 (Kelly data set) and PRJEB34332 (MiFish data set).
