# Supplementary material for: Mapping biodiversity hotspots of fish communities in subtropical streams through environmental DNA
Source: Sci Rep. 2021 May 14;11:10375. doi: 10.1038/s41598-021-89942-6 (PMC8121892; doi:10.1038/s41598-021-89942-6)
Supplement: Supplementary file 1 — Supplementary information. [file 41598_2021_89942_MOESM1_ESM.pdf]

SUPPLEMENTARY INFORMATION

Title:

Mapping biodiversity hotspots of fish communities in subtropical streams through environmental DNA

Author list and ORCID ID:

Rosetta C. Blackman[a, 1, 2,3 *] - https://orcid.org/0000-0002-6182-8691

Maslin Osathanunkul[a, 1, 4, 5] - https://orcid.org/0000-0001-6154-2813

Jeanine Brantschen[1, 2] - https://orcid.org/0000-0002-2945-3607

Cristina Di Muri[6] - https://orcid.org/0000-0003-4072-0662

Lynsey R. Harper[6, 7] - https://orcid.org/0000-0003-0923-1801

Elvira Mächler[1, 2, 8] - https://orcid.org/0000-0003-0430-6173

Bernd Hänfling[6] - https://orcid.org/0000-0001-7630-9360

Florian Altermatt[1, 2, 3 *] - https://orcid.org/0000-0002-4831-6958

Author affiliation:

[1] Eawag, Swiss Federal Institute of Aquatic Science and Technology, Department of Aquatic Ecology, Überlandstrasse 133, CH-8600 Dübendorf, Switzerland

[2] Department of Evolutionary Biology and Environmental Studies, University of Zurich, Winterthurerstr. 190, CH-8057 Zürich, Switzerland

[3] Research Priority Programme Global Change and Biodiversity (URPP GCB), University of Zurich, Winterthurerstr. 190, CH-8057 Zürich, Switzerland

[4] Department of Biology, Faculty of Science, Chiang Mai University, 50200 Chiang Mai, Thailand

[5] Research Center in Bioresources for Agriculture, Industry and Medicine, Chiang Mai University, Thailand

[6] Evolutionary and Environmental Genomics Group (EvoHull), School of Biological and Marine Sciences, University of Hull, Hull, HU6 7RX, United Kingdom

[7] School of Biological and Environmental Sciences, Liverpool John Moores University, Liverpool, L3 3AF, United Kingdom

[8] Centre for Fish and Wildlife Health, Department for Infectious Diseases and Pathobiology, Vetsuisse Faculty, University of Bern, Länggassstrasse 122, 3012 Bern, Switzerland

Equal contributions: [a] – joint first authorship

Corresponding Authors: [*] - To whom correspondence may be addressed:

Florian Altermatt, Department of Evolutionary Biology and Environmental Studies, University of Zurich, Winterthurerstr. 190, CH-8057 Zürich, Switzerland

Phone: +41 (0)58 765 5592; E-mail: florian.altermatt@eawag.ch

Rosetta C. Blackman, Eawag, Swiss Federal Institute of Aquatic Science and Technology, Department of Aquatic Ecology, Überlandstrasse 133, CH-8600 Dübendorf, Switzerland

Email: rosiecblackman@gmail.com

Contents for info:

Figure S1: Sampling sites

Figure S2: Read distribution from each primer

Figure S3: Proportion of taxa found in each replicate

Figure S4: Catchment Richness accumulation curve

Figure S5: Dendrogram

Figure S6: Optimal Clustering Analysis

Figure S7: Environmental variables PCA

Table S1: Dummy sequences

Table S2: eDNA fish detection

Table S3: Northern Thailand curated sequence reference database

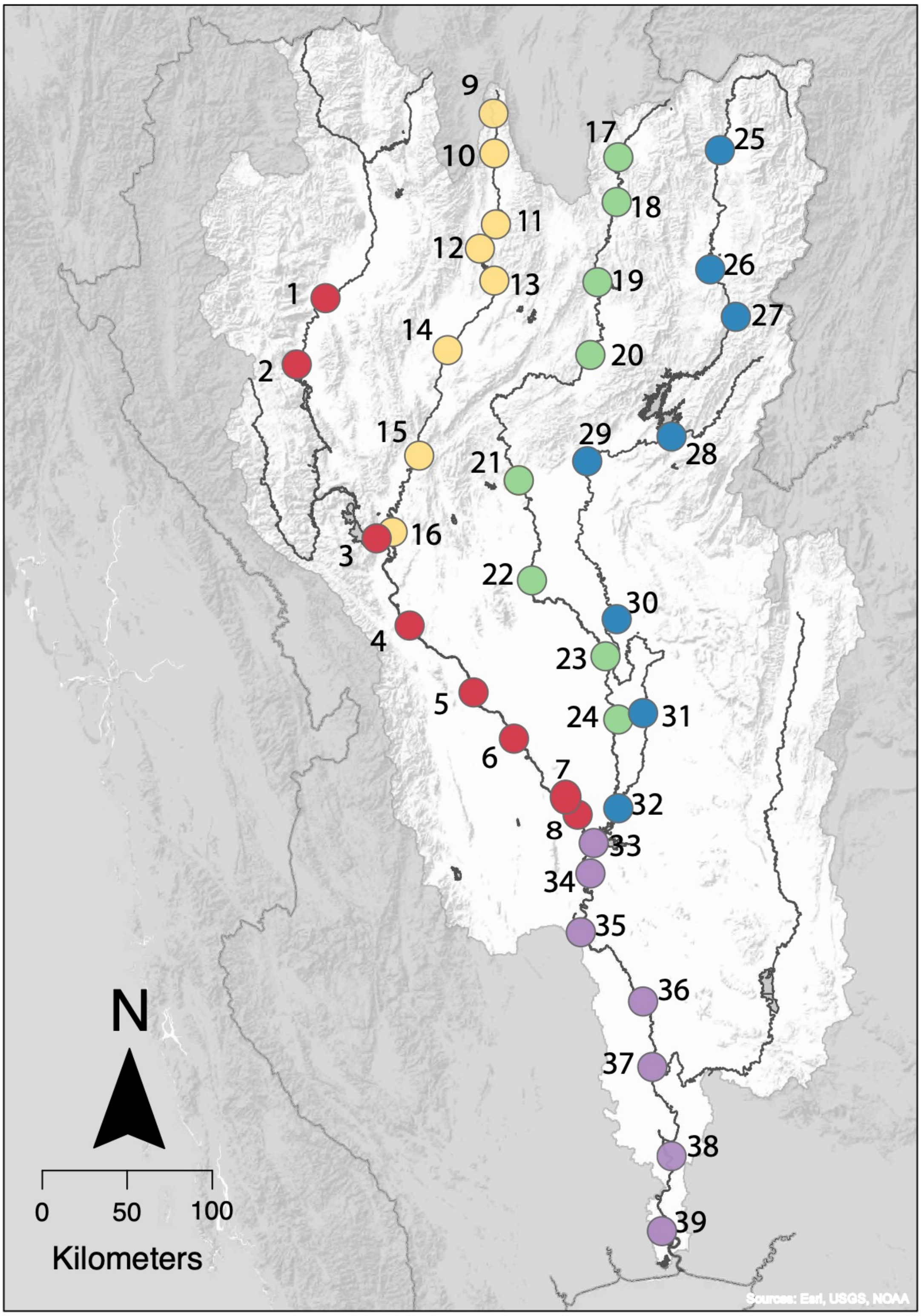

Figure S1: Sampling sites. 39 sites were sampled over the Chao Phraya catchment in Thailand for this study. The catchment (white background) is made up of 5 river sections: River Ping (red points), River Wang (yellow points), River Yom (green points), River Nan (blue points) and lower Chao Phraya (lilac points). The base map data was sourced from HydroSHEDS. 2015: WWF in partnership with USGS, CIAT, TNC, CESR: Esri, 2013 and mapped using ArcGIS, river colours were added using Adobe Illustrator version 25.2.1.

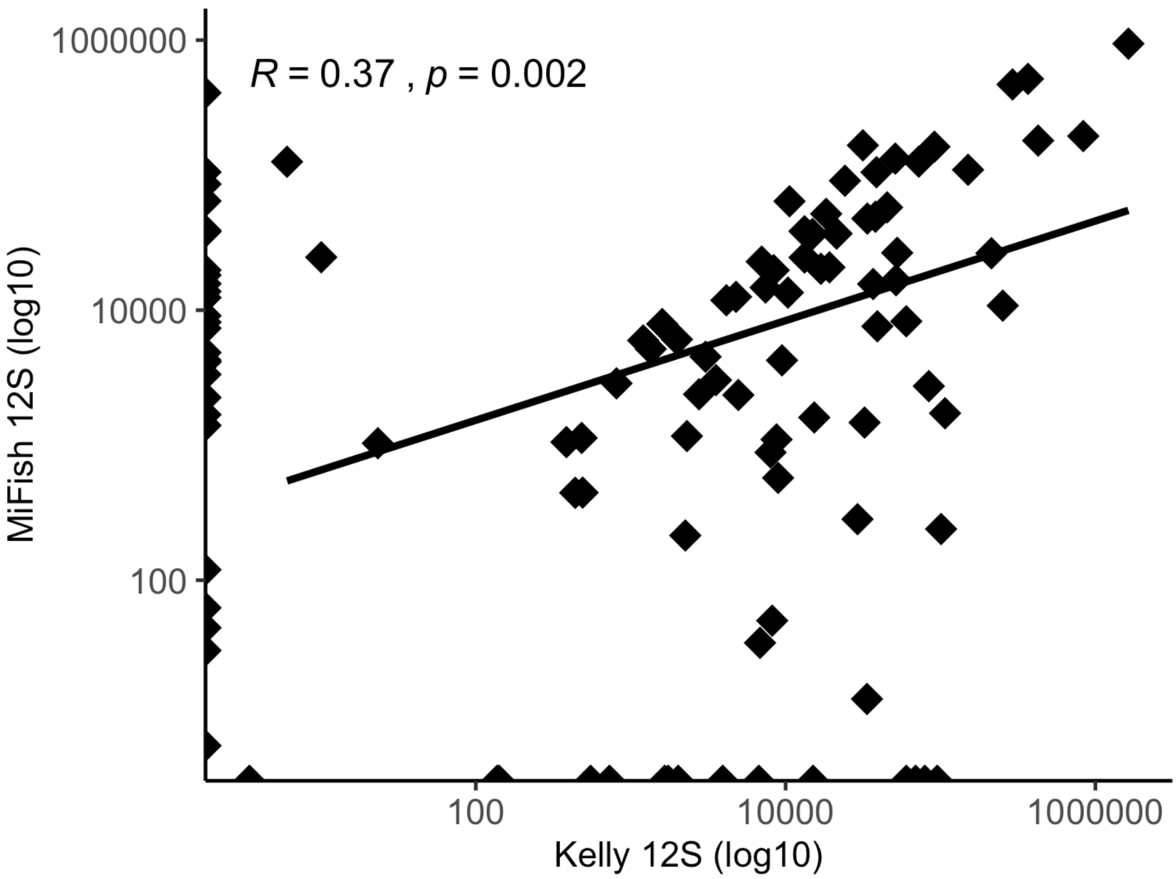

Figure S2: Read distribution from each primer. The combined read count of each fish taxon identified in this study by each of the two primer pairs: MiFish and Kelly.

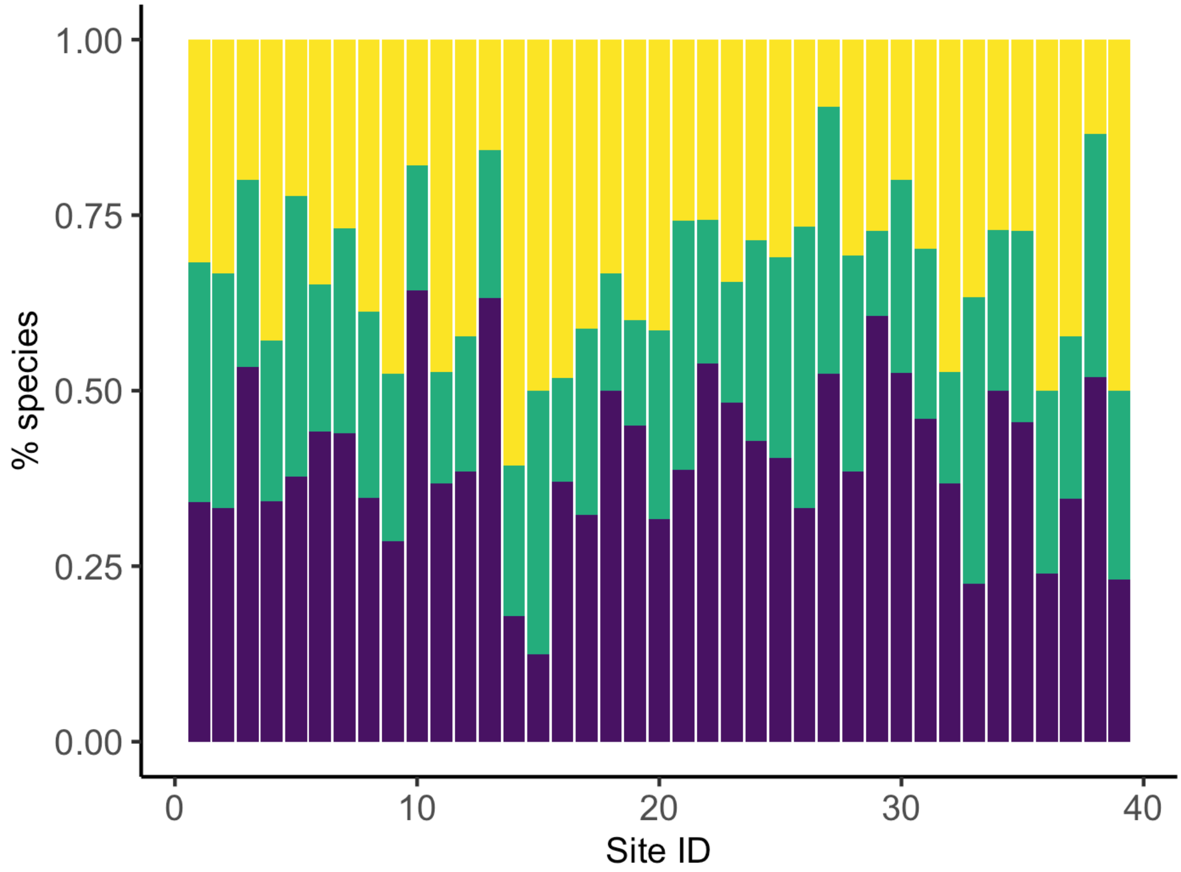

Figure S3: Proportion of fish taxa (FT) at each site found in each site replicate. At each site three replicates were collected (right bank, centre and left bank). Here the proportion of taxa found in 1, 2 or all 3 site replicates is shown: proportion of taxa found in only one site replicate (purple), proportion of taxa found in two site replicates (green) and taxa found in all three site replicates (yellow).

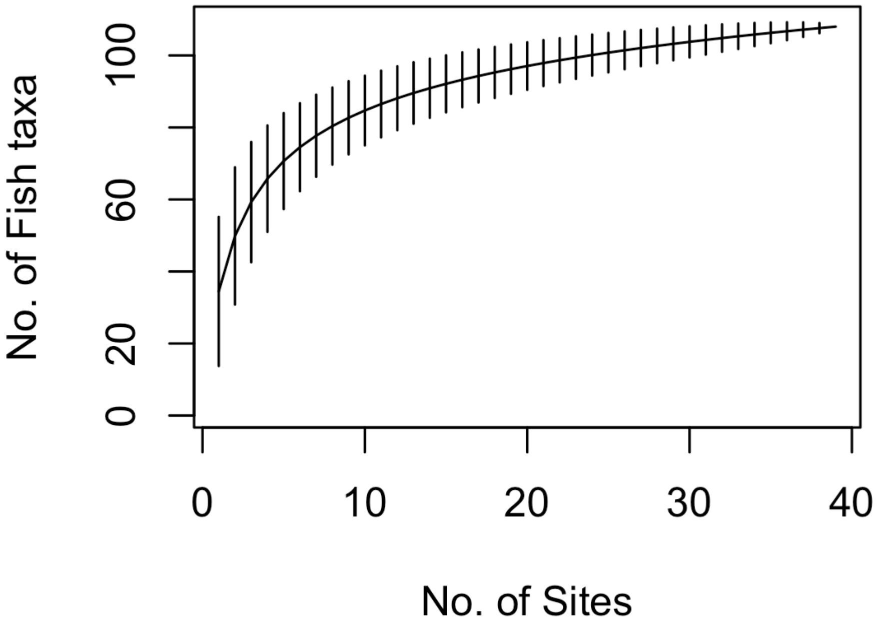

Figure S4: Catchment Richness accumulation curve. Total species richness accumulation for the catchment (39 samples).

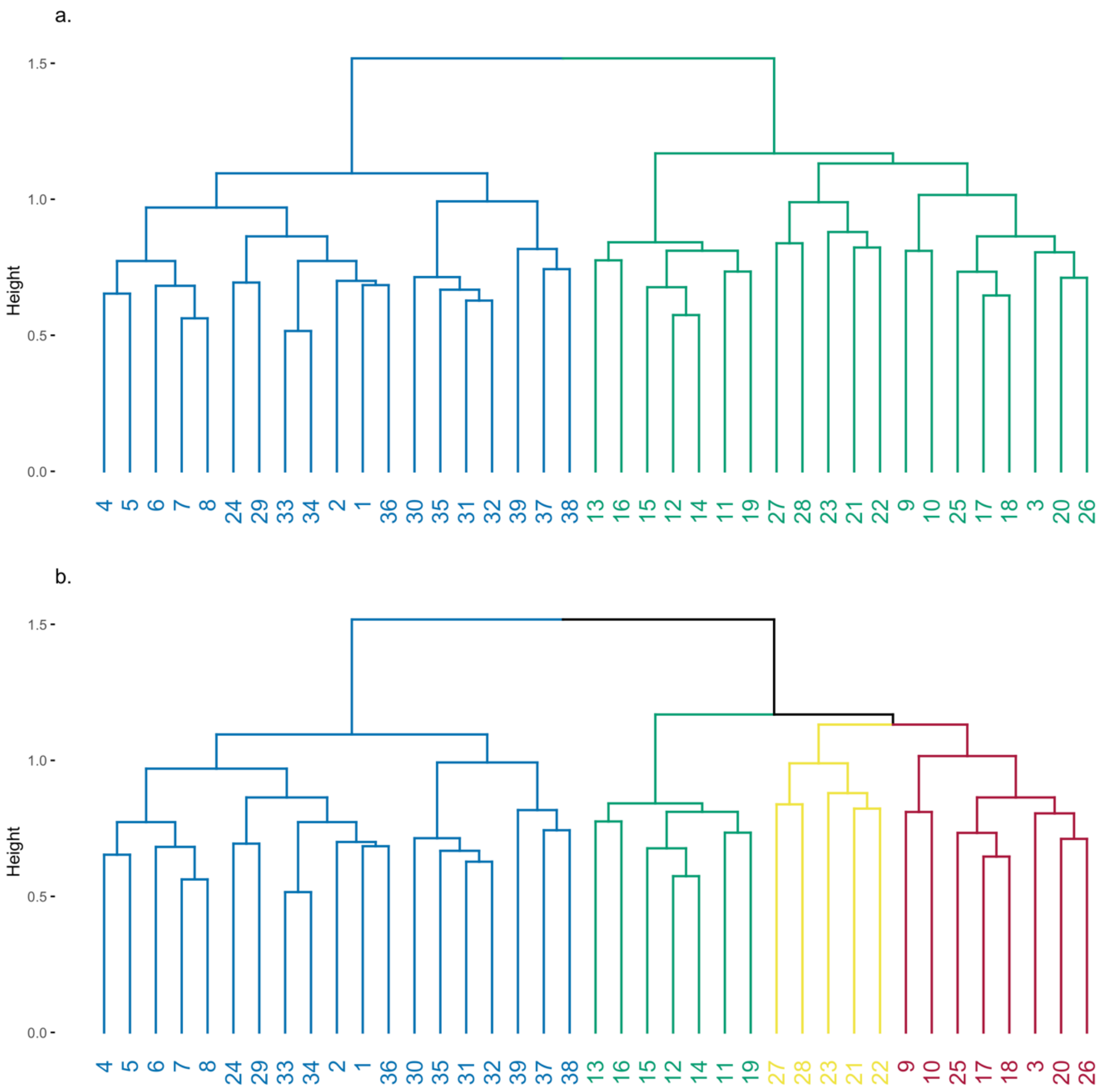

Figure S5: Dendrogram. Using hierarchical clustering with ward.D2 link function and Canberra distance we plotted 2 (a.) and 4 (b.) clusters as optimal clusters generated from the internal and stability validation analysis. Numbers at tips of dendrogram correspond to site IDs.

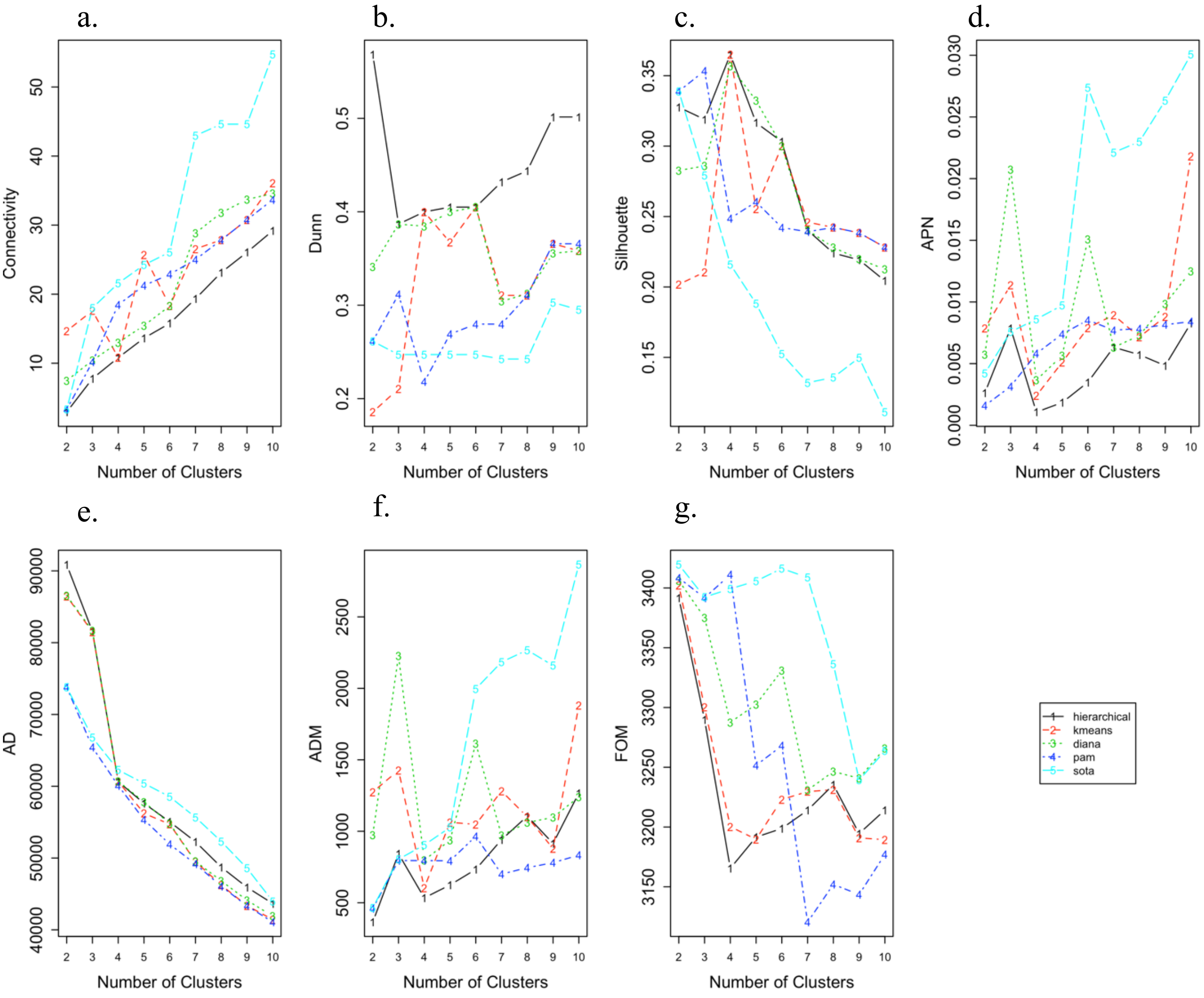

Figure S6: Optimal Clustering Analysis: Internal measures. a. Optimal clusters generated for connectivity: 2 clusters (2.9290) method hierarchical; b. Dunn Index: 2 clusters (0.5684) method hierarchical and c. Silhouette width: 4 clusters (0.3651) method hierarchical. **Stability measures:** d. Average proportion of non-overlap (APN) 4 clusters (0.0011) hierarchical, e. Average Distance (AD) 10 clusters (41069.6183) PAM clustering, f. Average Distance between means (ADM) 2 clusters (364.1612) hierarchical and g. Figure of Merit (FOM) 7 clusters (3120.4164) PAM clustering.

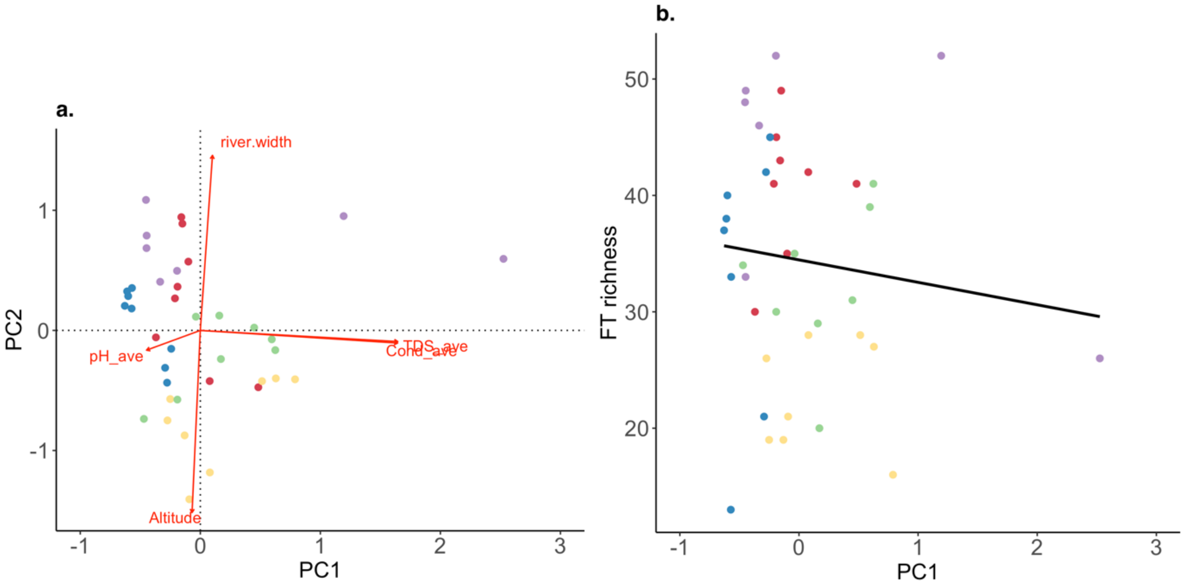

Figure S7: Environmental variables PCA. a. Principal component analysis (PCA) for the environmental variables (river width (m), Average pH, Average conductivity, Total dissolved solids and altitude (m)), (PC1: 40.79% and PC2: 32.4% of variance). **b.** PC1 plotted against FT richness at each site. The black line indicates the linear model, $R^2 = -0.11$, $p = 0.5$ (River colours correspond to Fig. S1).

Table S1: Dummy sequences: Artificially produced dsDNA based on customized sequence (GeneArt Strings, ThermoFisher) was used in the first PCR (1 µL, concentration 0.0001 ng/µL). Bold letters indicate primer binding sites and sequences are given in the 5'-3' direction.

Primer pair	Sequence
Kelly	**ACTGGGATTAGATACCCC**CTAATAGTTGTCGACAGATCGTCAAGATTAGAAAATGGTAGCAGCATTATCGGAGGTTCTCTAACTAGTATGGATAGCCGTGTCTTCACTGTGCTGCGGCTACCCATCGCCTGA**CTAGAGGAGCCTGTTCTA**
MiFish	**GTCGGTAAAACTCGTGCCAGC**ACCGGAAGACATCTCAGTTGAAGTGGTCTATACGACAGAGACCGTGCACCTACCAAATCTCCTTAGTGTAAGTTCAGACCAATTGGTAGTTTGTCCAGAACTCAGATTTTAACAGCAGAGGACGCATGCTCTATCTTTATGATCCATTGATGTCCCTGAGGCTGCAATATAT**CAAACTGGGATTAGATACCCCACTATG**

Table S2: eDNA fish detection: Details of the taxonomic detection by each of the two primer pairs tested in this study. Green denotes the taxa that were detected by the primer pair, orange denotes taxa that were not detected by the primer pair.

Species detected with eDNA	MiFish primer	Kelly primer
Acantopsis choirorhynchos	Yes	Yes
Amblyrhynchichthys truncatus	Yes	Yes
Anabas testudineus	Yes	Yes
Aperioptus gracilentus	Yes	Yes
Barbichthys laevis	Yes	Yes
Barbonymus altus	Yes	Yes
Barbonymus gonionotus	Yes	Yes
Barbonymus schwanefeldii	Yes	No
Boesemania microlepis	Yes	No
Cephalocassis borneensis	No	Yes
Channa gachua	Yes	Yes
Channa lucius	Yes	No
Channa micropeltes	Yes	Yes
Channa striata	Yes	Yes
Chitala ornata	No	Yes
Cirrhinus molitorella	Yes	No
Cirrhinus mrigala	Yes	No
Clarias batrachus	No	Yes
Clarias gariepinus	Yes	Yes
Clupeoides borneensis	Yes	Yes
Coilia lindmani	Yes	Yes
Cosmochilus harmandi	Yes	Yes
Crossocheilus reticulatus	Yes	No
Cyclocheilichthys apogon	Yes	No
Cyclocheilichthys enoplos	Yes	Yes
Cyprinus carpio	Yes	Yes
Danio albolineatus	No	Yes
Dermogenys pusilla	Yes	No
Discherodontus schroederi	Yes	No
Doryichthys boaja	Yes	Yes
Epalzeorhynchos bicolor	No	Yes
Esomus metallicus	Yes	Yes
Eugnathogobius siamensis	Yes	Yes
Gambusia affinis	Yes	No
Garra cambodgiensis	Yes	No
Gyrinocheilus aymonieri	Yes	No
Hampala macrolepidota	Yes	Yes

Hemibagrus wyckii	Yes	Yes
Hemibagrus wyckioides	Yes	Yes
Henicorhynchus lineatus	Yes	Yes
Henicorhynchus siamensis	Yes	No
Homaloptera smithi	No	Yes
Homalopteroides smithi	Yes	Yes
Hypophthalmichthys molitrix	Yes	Yes
Hypophthalmichthys nobilis	Yes	Yes
Hypostomus plecostomus	Yes	Yes
Hypsibarbus malcolmi	Yes	Yes
Ictalurus punctatus	Yes	Yes
Labeo chrysophekadion	Yes	No
Labeo rohita	Yes	Yes
Labiobarbus leptocheilus	No	Yes
Labiobarbus siamensis/leptocheilus	Yes	No
Leptobarbus hoevenii	Yes	No
Lobocheilos melanotaenia	No	Yes
Luciosoma bleekeri	Yes	Yes
Lycothrissa crocodilus	Yes	Yes
Macrognathus siamensis	Yes	No
Mastacembelus armatus	Yes	Yes
Mystacoleucus marginatus	Yes	Yes
Mystus atrifasciatus	No	Yes
Mystus singaringan	Yes	Yes
Nemacheilus pallidus	Yes	Yes
Nemacheilus platiceps	Yes	No
Notopterus notopterus	Yes	Yes
Opsarius koratensis	Yes	No
Opsarius pulchellus	Yes	Yes
Oreochromis sp.	Yes	Yes
Osphronemus goramy	Yes	Yes
Osteochilus melanopleurus	Yes	Yes
Osteochilus microcephalus	Yes	No
Osteochilus waandersii	Yes	No
Oxyeleotris marmorata	Yes	Yes
Pangasianodon gigas	No	Yes
Pangasianodon hypophthalmus	Yes	Yes
Pangasius larnaudii	Yes	Yes
Pangasius macronema	Yes	Yes
Pangio anguillaris	Yes	Yes
Pangio oblonga	No	Yes
Parachela siamensis	Yes	Yes

Paralaubuca typus	Yes	Yes
Pethia ticto	Yes	Yes
Polynemus dubius	Yes	Yes
Poropuntius bantamensis	Yes	No
Pristolepis fasciata	Yes	Yes
Pseudolais pleurotaenia	Yes	Yes
Pseudomystus siamensis	Yes	Yes
Puntigrus partipentazona	No	Yes
Puntioplites proctozystron	Yes	No
Puntius brevis	Yes	Yes
Raiamas guttatus	Yes	Yes
Rasbora borapetensis	No	Yes
Rasbora dusonensis	Yes	Yes
Rasbora paviana	Yes	Yes
Rhinogobius chiengmaiensis	Yes	No
Scaphiodonichthys acanthopterus	Yes	No
Setipinna melanochir	No	Yes
Syncrossus helodes	Yes	Yes
Thynnichthys thynnoides	Yes	Yes
Tor tambroides	Yes	Yes
Toxotes chatareus	Yes	Yes
Trichopodus pectoralis	Yes	Yes
Trichopodus trichopterus	Yes	Yes
Wallago attu	No	Yes
Yasuhikotakia eos	Yes	Yes
Yasuhikotakia lecontei	Yes	Yes
Yasuhikotakia modesta	Yes	Yes
Yasuhikotakia morleti	Yes	Yes
Yasuhikotakia sidthimunki	Yes	No

Table S3: Northern Thailand curated sequence reference database: Details of the fish species found in Thailand and the sequences included in the reference database for this study. Green denotes species sequences included in the curated database and the number of sequences included. Orange denotes no sequence available for that primer pair.

Species list Northern Thailand	Present in Database	# MiFish sequences	# Kelly sequences
Acanthopsoides gracilis	Yes		1
Acantopsis choirorhynchos	Yes	1	1
Acantopsis dialuzona	Yes	1	
Acantopsis rungthipae	Yes	1	
Acantopsis thiemmedhi	Yes	1	
Acentrogobius viridipunctatus	Yes	1	1
Albulichthys albuloides	Yes	1	1
Allenbatrachus grunniens	Yes	2	1
Ambastaia sidthimunki (Yasuhikotakia sidthimunki)	Yes	3	3
Amblyceps mangois	Yes	1	
Amblypharyngodon chulabhornae	Yes	2	2
Amblyrhynchichthys truncatus	Yes	1	1
Anabas testudineus	Yes	2	3
Anematichthys apogon (Cyclocheilichthys apogon)	Yes	2	
Anematichthys repasson	Yes	1	
Anguilla japonica	Yes	5	6
Aperiptus gracilentus	Yes	1	1
Aplocheilus panchax	Yes	1	2
Arius maculatus	Yes	1	1
Bagarius bagarius	Yes	1	1
Bagarius yarrelli	Yes	2	2
Bagrichthys obscurus	Yes		1
Balantiocheilos melanopterus	Yes	1	1
Barbichthys laevis	Yes	1	1
Barbodes aurotaeniatus	Yes	1	1
Barbodes binotatus	Yes	2	1
Barbonymus altus	Yes	1	1
Barbonymus gonionotus	Yes	2	2
Barbonymus schwanenfeldii	Yes	2	2
Betta smaragdina	Yes		1
Boesemania microlepis	Yes	1	
Boleophthalmus boddarti	Yes	1	1
Boraras urophthalmoides	Yes	1	
Brachirus harmandi	Yes	1	

Caragobius urolepis	Yes	1	
Carassius auratus	Yes	18	17
Carinotetraodon lorteti	Yes	1	1
Catlocarpio siamensis	Yes	1	1
Cephalocassis borneensis	Yes		1
Channa gachua	Yes	2	2
Channa lucius	Yes	1	1
Channa micropeltes	Yes	1	1
Channa striata	Yes	2	2
Chitala lopis	Yes	1	1
Chitala ornata	Yes	1	2
Cirrhinus microlepis	Yes	1	1
Cirrhinus molitorella	Yes	3	2
Cirrhinus mrigala	Yes	2	3
Clarias batrachus	Yes	2	2
Clarias gariepinus	Yes	2	2
Clupeoides borneensis	Yes	1	1
Coilia lindmani	Yes	1	1
Cosmochilus harmandi	Yes	1	1
Crossocheilus atrilimes	Yes	3	3
Crossocheilus reticulatus	Yes	2	2
Cryptarius truncatus	Yes		1
Ctenopharyngodon idella	Yes	5	4
Cyclocheilichthys enoplos	Yes	1	1
Cyprinus carpio	Yes	11	15
Danio albolineatus	Yes	2	2
Datnioides microlepis	Yes	2	1
Dermogenys pusilla	Yes	1	2
Discherodontus schroederi	Yes	1	1
Doryichthys boaja	Yes	2	2
Eirmotus octozona	Yes	1	1
Epalzeorhynchos bicolor	Yes	1	2
Epalzeorhynchos frenatus	Yes	1	2
Epalzeorhynchos munense	Yes	1	1
Esomus metallicus	Yes	1	2
Eugnathogobius siamensis	Yes	1	
Gambusia affinis	Yes	1	3
Garra cambodgiensis	Yes	1	
Garra fasciacauda	Yes	1	
Glossogobius aureus	Yes	1	1
Glyptothorax laosensis	Yes	1	1
Glyptothorax trilineatus	Yes	1	1

Gobiopterus chuno	Yes	1	1
Gyrinocheilus aymonieri	Yes	4	4
Hampala dispar	Yes	1	1
Hampala macrolepidota	Yes	3	2
Helostoma temminckii	Yes	1	1
Hemiarius stormii	Yes		1
Hemibagrus filamentus	Yes		1
Hemibagrus nemurus	Yes		1
Hemibagrus wyckii	Yes	1	2
Hemibagrus wyckioides	Yes	1	1
Henicorhynchus lineatus	Yes	1	1
Henicorhynchus lobatus	Yes	1	1
Henicorhynchus siamensis	Yes	2	2
Heteropneustes fossilis	Yes	4	4
Homalopteroides smithi	Yes	1	1
Hypophthalmichthys molitrix	Yes	18	8
Hypophthalmichthys nobilis	Yes	13	12
Hypostomus plecostomus	Yes	1	1
Hypsibarbus malcolmi	Yes	1	1
Hypsibarbus vernayi	Yes	1	1
Ictalurus punctatus	Yes	6	6
Incislabeo behri (Bangana behri)	Yes	1	
Indostomus paradoxus	Yes	2	1
Kryptopterus bicirrhis	Yes	1	1
Labeo chrysophekadion	Yes	2	1
Labeo dyocheilus	Yes	1	1
Labeo pierrei	Yes	2	1
Labeo rohita	Yes	3	5
Labiobarbus leptocheilus	Yes	2	2
Labiobarbus siamensis	Yes	1	
Leiognathus ruconius	Yes	1	
Lepidocephalichthys berdmorei	Yes		1
Lepidocephalichthys hasselti	Yes	1	1
Lepidocephalichthys micropogon	Yes	1	1
Leptobarbus hoevenii	Yes	1	1
Lobocheilos bo	Yes	1	1
Lobocheilos melanotaenia	Yes	1	1
Luciosoma bleekeri	Yes	1	1
Luciosoma setigerum	Yes	1	1
Lycothrissa crocodilus	Yes	1	1
Macrochirichthys macrochirus	Yes	2	
Macrognathus aculeatus	Yes	2	3

Macrognathus siamensis	Yes	1	
Mastacembelus armatus	Yes	2	3
Mastacembelus erythrotaenia	Yes	1	2
Mastacembelus favus	Yes	1	1
Megalops cyprinoides	Yes	1	1
Monopterus albus	Yes	4	6
Mystacoleucus marginatus	Yes	3	3
Mystus atrifasciatus	Yes	2	2
Mystus bocourti	Yes		2
Mystus cavasius	Yes	2	1
Mystus gulio	Yes		1
Mystus multiradiatus	Yes		1
Mystus rhegma	Yes		2
Mystus singaringan	Yes	1	2
Nandus oxyrhynchus	Yes		1
Nemacheilus pallidus	Yes	1	1
Nemacheilus platiceps	Yes	1	1
Neolissochilus stracheyi	Yes	1	5
Notopterus notopterus	Yes	2	4
Ompok bimaculatus	Yes	1	11
Onychostoma gerlachi	Yes	1	1
Opsarius koratensis	Yes	1	
Opsarius pulchellus	Yes	1	2
Oreichthys cosuatis	Yes	1	1
Oreochromis aureus	Yes	2	2
Oreochromis mossambicus	Yes	1	1
Oreochromis niloticus	Yes	3	6
Oryzias minutillus	Yes	1	2
Osphronemus goramy	Yes	1	2
Osteochilus melanopleurus	Yes	1	1
Osteochilus microcephalus	Yes	1	
Osteochilus schlegelii	Yes	1	1
Osteochilus waandersii	Yes	1	1
Oxyeleotris marmorata	Yes	4	5
Pangasianodon gigas	Yes	1	1
Pangasianodon hypophthalmus	Yes	2	1
Pangasius bocourti	Yes		2
Pangasius conchophilus	Yes		2
Pangasius larnaudii	Yes	1	3
Pangasius macronema	Yes	1	2
Pangasius pangasius	Yes	2	2
Pangasius sanitwongsei	Yes		1

Pangio anguillaris	Yes	1	2
Pangio oblonga	Yes	1	2
Pao palembangensis	Yes	2	2
Parachela oxygastroides	Yes	1	
Parachela siamensis	Yes	1	1
Paralaubuca barroni	Yes		1
Paralaubuca typus	Yes	1	1
Parambassis wolffii	Yes		1
Pennahia argentata	Yes	1	1
Pethia ticto	Yes	2	3
Phenacostethus smithi	Yes	1	
Pisodonophis cancrivorus	Yes	3	2
Poecilia reticulata	Yes	3	5
Polynemus dubius	Yes	1	1
Poropuntius bantamensis	Yes	1	1
Pristolepis fasciata	Yes	1	1
Probarbus jullieni	Yes	1	1
Pseudolais pleurotaenia	Yes	1	2
Pseudomystus siamensis	Yes	1	2
Puntigrus partipentazona	Yes	1	1
Puntioplites proctozystron	Yes	1	1
Puntius brevis	Yes	1	1
Raiamas guttatus	Yes	1	1
Rasbora argyrotaenia	Yes	1	1
Rasbora borapetensis	Yes	1	1
Rasbora daniconius	Yes	2	2
Rasbora dusonensis	Yes	1	1
Rasbora paviana	Yes	1	1
Rasbora sumatrana	Yes	1	1
Rasbora trilineata	Yes	2	3
Rhinogobius chiengmaiensis	Yes	1	
Rhinogobius mekongianus	Yes	1	
Scaphiodonichthys acanthopterus	Yes	1	1
Scaphiodonichthys burmanicus	Yes	1	1
Schistura geisleri	Yes	1	1
Schistura pridii	Yes	1	1
Setipinna melanochir	Yes	2	1
Sikukia gudgeri	Yes	3	3
Sillago sihama	Yes	2	3
Syncrossus helodes	Yes	2	2
Syncrossus hymenophysa	Yes	2	2
Tachysurus nudiceps	Yes	1	

Tenualosa toli	Yes	1	1
Thynnichthys thynnoides	Yes	1	1
Tor douronensis	Yes	1	2
Tor tambroides	Yes	2	4
Toxotes chatareus	Yes	1	1
Toxotes microlepis	Yes		1
Trichopodus pectoralis	Yes	2	2
Trichopodus trichopterus	Yes	1	3
Trichopsis pumila	Yes		1
Trichopsis vittata	Yes		1
Tuberoschistura baenzigeri	Yes	1	1
Wallago attu	Yes	2	2
Yasuhikotakia eos	Yes	3	5
Yasuhikotakia lecontei	Yes	1	1
Yasuhikotakia modesta	Yes	2	2
Yasuhikotakia morleti	Yes	3	3
Pastinachus sephen	Yes	1	2
Pristis microdon	Yes	1	1
Acanthopsoides delphax	No		
Acanthopsoides hapalias	No		
Acrochordonichthys gyrinus	No		
Akysis maculipinnis	No		
Akysis recavus	No		
Amblyrhynchichthys micracanthus	No		
Bagarius suchus	No		
Bagrichthys hypselopterus	No		
Bagrichthys macracanthus	No		
Bagrichthys macropterus	No		
Bagroides melapterus	No		
Balantiocheilos ambusticauda	No		
Balitoropsis zollingeri	No		
Bangana ariza	No		
Bangana behri	No		
Bangana sinkleri	No		
Barbodes rhombeus	No		
Barbonymus balleroides	No		
Barilius huahinensis	No		
Barilius infrafasciatus	No		
Barilius ornatus	No		
Belodontichthys dinema	No		
Belodontichthys truncatus	No		
Brachirus panoides	No		

Brachirus siamensis	No		
Brachygobius sua	No		
Brachygobius xanthozonus	No		
Ceratoglanis pachynema	No		
Chaudhuria caudata	No		
Cirrhinus caudimaculatus	No		
Cirrhinus jullieni	No		
Clarias macrocephalus	No		
Clarias meladerma	No		
Colossoma bidens	No		
Corica soborna	No		
Crossocheilus cobitis	No		
Crossocheilus oblongus	No		
Crossocheilus tchangi	No		
Ctenogobius vexillifer	No		
Cyclocheilichthys armatus	No		
Cyclocheilichthys heteronema	No		
Cyclocheilichthys lagleri	No		
Cynoglossus microlepis	No		
Datnioides polota	No		
Datnioides pulcher	No		
Dermogenys siamensis	No		
Devario annandalei	No		
Devario maetaengensis	No		
Discherodontus halei	No		
Doryichthys deokhatoides	No		
Doryichthys martensii	No		
Esomus longimanus	No		
Exostoma effrenum	No		
Garra fisheri	No		
Garra fuliginosa	No		
Garra nasuta	No		
Garra waensis	No		
Glyptothorax buchanani	No		
Glyptothorax callopterus	No		
Glyptothorax fuscus	No		
Glyptothorax lampris	No		
Glyptothorax major	No		
Glyptothorax platypogonides	No		
Helicophagus leptorhynchus	No		
Helicophagus waandersii	No		
Hemiarius verrucosus	No		

Hemibagrus planiceps	No		
Hemimyzon nanensis	No		
Hemitrygon laosensis	No		
Heteropneustes kemratensis	No		
Hypsibarbus wetmorei	No		
Kryptopterus cheveyi	No		
Kryptopterus cryptopterus	No		
Kryptopterus dissitus	No		
Kryptopterus geminus	No		
Kryptopterus limpok	No		
Labeo indramontri	No		
Labeo yunnanensis	No		
Laubuka caeruleostigmata	No		
Laubuka laubuca	No		
Lepidocephalichthys furcatus	No		
Lepidocephalus nanensis	No		
Lobocheilos cryptopogon	No		
Lobocheilos delacouri	No		
Lobocheilos gracilis	No		
Lobocheilos nigrovittatus	No		
Lobocheilos quadrilineatus	No		
Lobocheilos rhabdoura	No		
Lobocheilos thavili	No		
Longiculter siahi	No		
Macrognathus circumcinctus	No		
Macrognathus maculatus	No		
Macrognathus semiocellatus	No		
Macrognathus taeniagaster	No		
Macrotrema caligans	No		
Mayaheros urophthalmus	No		
Micronema moorei	No		
Mystacoleucus chilopterus	No		
Mystacoleucus greenwayi	No		
Mystus albolineatus	No		
Mystus mysticetus	No		
Mystus nigriceps	No		
Mystus velifer	No		
Nandus nebulosus	No		
Nemacheilus binotatus	No		
Nemacheilus masyae	No		
Neolissochilus subterraneus	No		
Ompok eugeneiatus	No		

Ompok hypophthalmus	No		
Ompok pinnatus	No		
Opsarius bernatziki	No		
Oreoglanis colurus	No		
Oreoglanis nakasathiani	No		
Oreoglanis siamensis	No		
Oreoglanis sudarai	No		
Oreoglanis tenuicauda	No		
Oreoglanis vicinus	No		
Osteochilus lini	No		
Osteochilus vittatus	No		
Oxygaster pointoni	No		
Pangasius elongatus	No		
Pangio fusca	No		
Pao abei	No		
Pao cochinchinensis	No		
Pao leiurus	No		
Pao turgidus	No		
Paracanthocobitis mandalayensis	No		
Parachela maculicauda	No		
Parachela williaminae	No		
Paralaubuca harmandi	No		
Paralaubuca riveroi	No		
Paralaubuca stigmabrachium	No		
Parambassis baculis	No		
Parambassis siamensis	No		
Parambassis thomassi	No		
Pethia stoliczkana	No		
Phalacronotus apogon	No		
Phalacronotus bleekeri	No		
Phalacronotus micronemus	No		
Physoschistura pseudobrunneana	No		
Platytropius siamensis	No		
Polynemus aquilonaris	No		
Polynemus multifilis	No		
Poropuntius chondrorhynchus	No		
Poropuntius deauratus	No		
Poropuntius faucis	No		
Pseudobagarius hardmani	No		
Pseudobagarius leucorhynchus	No		
Pseudohomaloptera sexmaculata	No		
Pseudolais micronemus	No		

Puntius sophoroides	No		
Puntius spilopterus	No		
Rasbora aurotaenia	No		
Rasbora dorsinotata	No		
Rasbora einthovenii	No		
Rasbora rasbora	No		
Schistura breviceps	No		
Schistura bucculenta	No		
Schistura desmotes	No		
Schistura dubia	No		
Schistura kengtungensis	No		
Schistura kohchangensis	No		
Schistura maejotigrina	No		
Schistura menanensis	No		
Schistura nicholsi	No		
Schistura poculi	No		
Schistura reidi	No		
Schistura schultzi	No		
Schistura sexcauda	No		
Schistura sirindhornae	No		
Schistura spilota	No		
Schistura waltoni	No		
Sectoria atriceps	No		
Secutor ruconius	No		
Sikukia stejnegeri	No		
Systomus jacobusboehlkei	No		
Systomus rubripinnis	No		
Thryssocypris wongrati	No		
Trichopodus microlepis	No		
Wallago leerii	No		
Wallago micropogon	No		
Fluvitrygon oxyrhynchus	No		
Fluvitrygon signifer	No		
Himantura krempfi	No		
Himantura oxyrhyncha	No		
Himantura polylepis	No		
Himantura signifer	No		
Urogymnus polylepis	No		
